# Integrating continuous flow reaction and work-up: chiral amine resolution, separation and purification using a novel coalescing filter system†

**DOI:** 10.1039/d4re00442f

**Published:** 2024-11-13

**Authors:** Bethan M. Rowley, Lisa A. Thompson, Luke A. Power, James Daglish, Emma Parks, James Birbeck, Steve Marsden, Nikil Kapur, A. John Blacker

**Affiliations:** a Institute of Process Research and Development, Schools of Chemistry, Chemical and Process Engineering, Mechanical Engineering, University of Leeds Woodhouse Lane Leeds West Yorkshire UK j.blacker@leeds.ac.uk; b Croda Europe Ltd. Goole East Yorkshire UK

## Abstract

To maximize the benefits of a continuous flow reaction, a continuous work-up is also needed. Herein, we present a process design and novel equipment for a continuous amine resolution reaction, integrated with liquid–liquid (L–L) extraction, back-extraction into a different solvent, and crystallisation purification for product isolation. The reaction, in iso-propyl acetate, flows through a heated fixed-bed reactor with solid supported *Candida antarctica* lipase which catalyses the resolution of (*rac*)-1-phenylethylamine to give the (*R*)-amide in 50% conversion and 96% enantiomeric excess (ee). This is separated from the unreacted (*S*)-amine co-product by mixing with an acidic aqueous stream and separating the phases using our recently reported coalescence filter separator. The aqueous stream is neutralised by mixing with base and back-extracted into methyl-THF solvent before separating the phases using a membrane separator. Finally, a solid amine salt is isolated by filtration, achieved by mixing the free base with an organic acid to cause crystallisation to give the (*S*)-1-phenylethylamine in 43% yield and >99% ee from racemate. The work illustrates how typical reactions, work-up and purification steps that involve multiple phases can be telescoped together using both new and commercially available laboratory equipment. This continuous system uses mild reaction conditions, green solvents and minimises their use for reduced waste.

## Abstract 2

Continuous telescoped reaction, liquid–liquid separation and purification of an amine, using a novel coalescing droplet filtration system, pH-based extractions and crystallisation.

## Graphical abstract

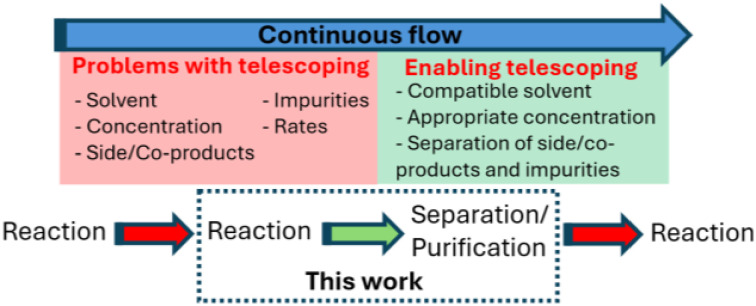

## Introduction

Over the past decade the continuous flow synthesis of fine chemicals and active pharmaceutical ingredients (APIs) for drug production has become increasingly widespread.^1–4^ Continuous flow systems offer a pathway to improved productivity, consistency and sustainability by intensification, improved control and reduced waste.^5–9^ The focus of much research is on homogeneous reactions using tubular reactors, with work-up and purification frequently done in batch.^10–13^ Several groups have recognised the need for laboratory scale equipment to carry out multi-phase reactions involving combinations of gas–liquid(s)–solid(s).^14,15^ A simple solution is to employ active mixing provided by continuous stirred tank reactors (CSTR), and there are an increasing number of examples of processes employing these.^16–19^ A current aim of many groups is to further improve process efficiency by joining or telescoping together two or more reactions by designing them to avoid work-up and isolation.^20–22^ Whilst this is possible in cases where a common reaction solvent is identified, there are many examples where this is not possible, and solvent exchange, separation of by-products, impurities and catalysts is required through work-up and purification. Many of the benefits of continuous reactions cannot be realised until downstream processes are also operated in a flow-mode.^23^ Operations that involve multiple phases, such as liquid–liquid (L–L) extraction, separation, distillation, crystallisation, and filtration, require laboratory-scale equipment of which there is little available. L–L separation systems have been described by several groups.^24–26^ Of these, the Zaiput membrane separator has had much beneficial impact, with numerous papers describing its use in a variety of L–L separations.^27–30^ Nevertheless, there are limitations of this equipment which include the useable range of phase ratios, incompatibility with partially water-miscible solvents, emulsions and surfactant containing systems, and temperature control.

**Fig. 1:**
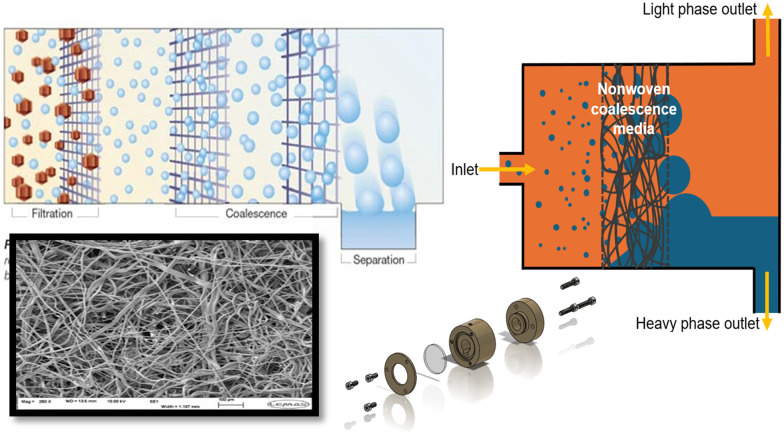
Anticlockwise from top left: principle of coalescence filtration; electron micrograph of PBT nonwoven filtration media; exploded view of coalescence filter housing; cross-section view of filter unit showing liquid flows.

Our work has sought to address some of these issues through the design of a coalescing filter employing nonwoven filter media, Fig. 1.^31^ The L–L separation mechanism differs from a cast polymeric membrane that separates at the leading surface; instead, the fibres cause the discrete liquid phase droplets to coalesce as they pass. The more-dense phase falls from the back of the media to create a separated phase that can be drawn from the bottom at a rate controlled by a valve/pump using the signal from conductance electrodes that infer the position liquid interface. The top layer flows out at a rate determined by the input phase volume. The use of nonwoven materials for coalescence filtration is particularly suited to act as a guard for solid capture.^32^ This is ideal for biotransformations where proteins and lipids cause fouling of cast membranes, leading to increased pressure difference and failure of liquid flow. Furthermore, the polybutylene terephthalate (PBT) media is produced in bulk for a wide variety of industries including automotive and medical.^33,34^

Biotransformations carried out in continuous flow are increasingly common with advantages that include reduced enzyme inhibition through continuous removal of products, easier downstream processing when using immobilised biocatalysts and improved total turnover numbers (TTNs).^35^ However, fast reaction rates are required to give short residence times, otherwise batch-mode may be more appropriate.^36^ Little research has been done on continuous flow biocatalytic technologies that combine synthesis and purification or work-up techniques.^2,13^ As an exemplar process, we report herein a continuous process for the synthesis of optically active amines with two consecutive purification steps, involving pH-based extractions and in-line crystallisation, to produce a solid product, using green solvents.

**Scheme 1:**
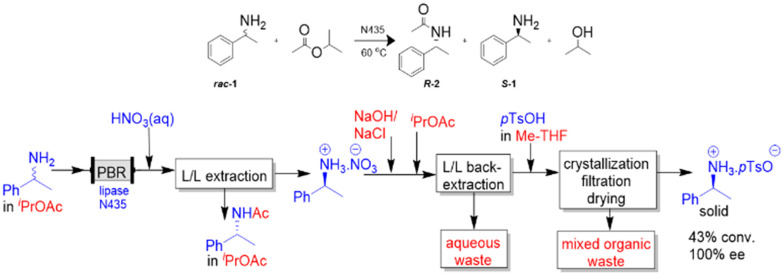
Continuous flow resolution of *rac*-1-phenylethylamine by enantioselective trans-acylation of isopropyl acetate with lipase N435, followed by acid extraction and separation, basification and back-extraction, acidification then batch crystallisation, filtration and drying to isolate solid (*S*)-1-phenylethylammonium tosylate product.

The immobilised lipase, Novozym435™ (N435) has previously been used in a continuous flow packed-bed reactor (PBR) for the ammonia amidation of (5*S*)-*N*-(*tert*-butoxycarbonyl)-5-(methoxycarbonyl)-2-pyrroline. The product was isolated in traditional batch manner by removal of solvent, residual ammonia, and isolated by recrystallisation from isopropanol in 96% yield and 98.5% purity.^37^ The same enzyme has also been used for the batch-mode resolution of primary amines by transacetylation.^38–40^ We decided to build on previous work to resolve chiral amines in continuous flow, and continuous selective L–L extraction of amines, by designing an integrated system for reaction followed by pH-mediated extraction, back-extraction and crystallisation Scheme 1.^28,41^

## Experimental

### Materials and methods

All the following compounds were purchased from suppliers and used without further purification. *rac*-1-Phenylethylamine (99%), biphenyl (99%), sodium hydroxide (NaOH, pellets), sodium chloride (NaCl, extra pure), *p*-toluenesulfonic acid monohydrate (*p*TsOH, 99%, extra pure) and 2-methyltetrahydrofuran (2-MeTHF, >99.5%) were purchased from Fisher Scientific Ltd. Iso-propyl acetate (98%) and nitric acid (HNO_3_, 69%) were purchased from Merck Life Science UK Ltd. Aqueous solutions were made up to required concentrations using ultrapure water. *Candida antartica* lipase supported on acrylic resin (≥5000 U g^−1^, recombinant, expressed in *Aspergillus niger*) also known as N435 was purchased from Scientific Laboratory Supplies. All analysis was performed using a HP 6890 GC system fitted with a CP-Chirasil Dex CB column.

**Scheme 2:**
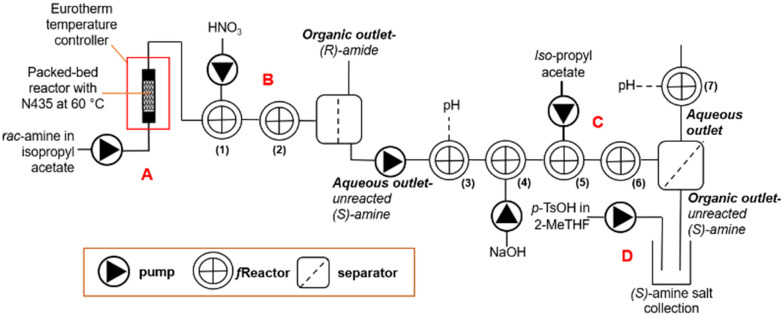
Equipment set-up for the process shown in Scheme 1. Showing: A. packed-bed reactor with lipase N435; B. acid-extraction, L–L separation and basification; C. back-extraction, L–L separation; D. re-acidification and crystallisation.

The general platform follows that shown in Scheme 2. *rac*-1-Phenylethylamine (*rac*-1) (0.07 M) in iso-propyl acetate was pumped using a JASCO PU-980 dual piston HPLC pump into a PBR (6.7 mL, 1.12 mL min^−1^) made from Swagelok stainless steel tubing (0.5′′ OD, 0.37′′ ID), filled with N435 (∼2 g) and glass beads (0.5 mm, ∼2 g). The PBR was connected to Swagelok SS810-R4 and SS-400-6-1ZV reducers and encased in a bespoke aluminium heating block and heated with a Eurotherm 3200 temperature controller at 60 °C, Scheme 2 Part A (see ESI† 1.2). The resultant product stream flowed into a stirred fReactor(1) CSTR, into which HNO_3_ (0.05 M) was pumped (1.12 mL min^−1^) using a second JASCO PU-980 pump, with the output flowing into fReactor(2) for additional mixing and extraction, as shown in Scheme 2.^18^ The biphasic reaction mixture was separated using a L–L coalescing separator fitted with 4 layers of PBT nonwoven media (see ESI† 1.1).^31^ The organic phase containing the (*R*)-amide (*R*-2) was collected whilst the aqueous phase was pumped using a KNF FEM 1.02 TTSM-2 pump into fReactor(3) (no stir bar) fitted with a Hanna Instruments HI-14132B pH probe, Scheme 2 Part B (see ESI† 1.1). This flowed into fReactor(4) and was mixed with a solution of NaOH (2 M)/NaCl (0.5 M) pumped (1.12 mL min^−1^) using a JASCO PU-980 pump to neutralise the unreacted (*S*)-amine (*S*-1) which was mixed and extracted into iso-propyl acetate pumped (1.12 mL min^−1^) using a Harvard apparatus model 11 syringe pump in fReactor(5); the biphasic mixture was mixed further in fReactor(6). For convenience, the biphasic reaction mixture was separated using a Zaiput technologies® SEP-10 L–L separator fitted with an O/B 900 membrane and the resultant aqueous phase flowed into fReactor(7) fitted with a Hanna Instruments HI-14132B pH probe, Scheme 2 Part C.

The amine containing organic phase was routed into a product collection vessel, into which a solution of *p*-TsOH (0.04 M) in 2-MeTHF was pumped (1.12 mL min^−1^) using a Harvard apparatus model 11 syringe pump. The resultant amine *p*-toluenesulfonate salt was collected by vacuum filtration to obtain the product, Scheme 2 Part D.

## Results and discussion

The chosen lipase is selective toward the (*R*)-enantiomer to produce the (*R*)-amide, leaving the (*S*)-amine unreacted, Scheme 1. Previous investigations focused on identifying a suitable acyl donor for the N435 lipase catalysed resolution of *rac*-1-phenylethylamine, Table 1.

**Table 1:** Performance of acyl donors in the resolution of *rac*-1-phenylethylamine^a^

Entry	Acyl donor	Time (h)	Conv^b^ (%)	Amide ee^c^ (%)	Amine ee^d^ (%)
1	Ethyl acetate	24	30	99	42
2	Iso-propyl acetate	24	50	99	99
3	Vinyl acetate	3.5	19	43	10
4	Iso-propenylacetate	30	38	86	53
5	*tert*-Butyl acetate	24	0	0	0
6	Methyl methoxyacetate	24	50	99	99
7	Ethyl methoxyacetate	24	49	99	97

a Reaction conditions: 0.07 mmol amine. 1 equiv. acyl donor, 10 mg lipase N435, 10 mL toluene 60 °C.

b Determined using conversion = [ee_amine_/(ee_amine_ + ee_amide_)] × 100.

c Determined by chiral GC analysis.

d Determined by chiral GC analysis after derivatisation using trifluoroacetic anhydride.^41^

Iso-propyl acetate outperformed ethyl acetate, entries 1 and 2, and performed as well as methyl and ethyl methoxyacetate, entries 6 and 7. The acyl donors, entries 3–5 performed less well. Based on this, iso-propyl acetate was selected as the preferred acyl donor.^41^ Furthermore, it also provides a better alternative to toluene as a green reaction and separation solvent.^42^ A series of continuous flow experiments were performed to determine the optimum residence time (*t*_res_), from 3–30 min, to find the highest conversion and enantioselectivity (see ESI† 1.3). A *t*_res_ of 6 min gave the highest conversion (44% (*R*)-amide from *rac*-1) and enantioselectivity (99% ee), so was used in the consecutive steps. The optimum steady-state reaction temperature was determined by incrementally changing, in 10-degree steps, the set-point of the electrically heated jacket from 30–100 °C. The optimum was 60 °C, giving steady state after 4 reactor volumes (RV) of the volume of the PBR.^41^ To maximise the productivity of the process a range of amine concentrations, from 0.07 to 0.7 M, were tested. At the lowest concentration a 49% conversion of *rac*-amine was seen, leaving unreacted (*S*)-amine of 96% ee, whilst at the highest concentration, only 42% conversion was seen, leaving (*S*)-amine of only 73% ee. The equivalent productivities are 41 and 355 g L^−1^ h^−1^, however since an enantiopure amine product is the aim of the resolution, the lower concentration was used for subsequent work.^41^ If further development work were done, a larger PBR could be used to increase the conversion at higher substrate concentration. Under these conditions the continuous reaction was run for 296 RV (30 hours) and for 240 RV (24 hours) (see ESI† 1.4) without change in the product conversion, whilst maintaining high ee, showing how stable the supported enzyme is. The substrate scope was tested with *rac*-4′-fluoro-1-phenylethylamine and *rac*-4′-phenyl-2-butylamine in continuous-mode, using the conditions described in Table 1, entry 6. The fluorinated amine gave, at steady-state, a perfect resolution of 50% conversion and 100% ee amide and amine, whilst the latter amine gave 47% conversion, 100% ee amide and 95% ee amine. This shows the potential for continuous resolution of more industrially relevant primary amines. The secondary amine *rac-N*-iso-propyl-1-phenylethylamine failed to react.^41^

Following the continuous biotransformation, the work-up required separation of the (*R*)-amide from unreacted (*S*)-amine. This was achieved by adding acid to extract the (*S*)-amine into water, leaving the (*R*)-amide in the iso-propyl acetate organic phase, Scheme 1. The concentration and feed rate of acid required to effect complete amine extraction was determined in continuous mode by varying the concentration of hydrochloric acid between 0.1 and 1 M at 1 : 1 phase ratio (0.5 ml min^−1^ each) using two fReactors to achieve complete amine mass transfer. To compare their performance, L–L separation was done using either the Zaiput or coalescence separator. The L–L coalescing separator was fitted with 4 layers of PBT filter and the Zaiput with a PTFE hydrophobic membrane (0.9 μm pore size). Residual amine in the organic phase was analysed (off-line), using biphenyl as the internal standard (Scheme 2 Part B). The acid concentration, was compared to the aqueous extraction efficiency defined as:^43^Extraction Efficiency = 1/(1 + *K*_D_)where *K*_D_ is the distribution coefficient of the amine across the two phases: *K*_D_ = *C*_Organic_/*C*_Aqueous_and *C* is the concentration of a compound in either the organic or aqueous phase. A linear increase in extraction efficiency was observed until 0.9 M acid where the amine was fully protonated and extracted (see ESI† 3.2). Given the amine is 0.035 M, this represents a 25-fold excess over the stoichiometric quantity and reflects the acidity required to protonate the amine (p*K*_α_ 10). The L–L coalescing separator allowed for complete separation of the two phases across all acid concentrations, whereas the Zaiput membrane separator suffered sporadic pressure rises due to fouling on the hydrophobic membrane, likely caused from precipitated salts, causing incomplete separation of the two phases. Furthermore, where downstream pressure changes were encountered, phase cross-over between the outlet streams was observed when using the Zaiput in Scheme 2, Part B, and whilst an intermediate tank and pump could have been used to avoid pressure fluctuations, this was less satisfactory than using the coalescing separator which tolerates downstream pressure. Therefore, the L–L coalescing separator brings benefit of operation where solid or debris is present that fouls the surface, and downstream conditions may vary. Further research testing the robustness of the L–L coalescing separator in relation to liquid–liquid extractions containing particulates is currently underway. Whilst hydrochloric acid was used in the extraction tests, it was switched to nitric acid for further work as it is more compatible with the stainless-steel pump heads employed. Being of similar acidity, it was added at the same concentration and rate, to give a measured pH of 1.4.

For the back-extraction, (Scheme 2 Part C), the equipment was assembled, and several points were identified: a pump was required to add NaOH to release the unreacted (*S*)-amine; the ionic strength was increased with NaCl to improve separation in the back-extraction; an additional pump was also needed to charge fresh iso-propyl acetate to extract the unreacted (*S*)-amine.

The base and solvent feeds were mixed with the aqueous (*S*)-amine using two fReactors in series (Scheme 2 Part C). The coalescing filter effected complete separation throughout the study. These conditions allowed the conductance electrode to identify the interface and actuate the pump to release the lower aqueous phase.^31^ The (*S*)-amine was extracted quantitatively, however, there was a 6% loss of (*R*)-amide from the organic phase measured *via* GC, due to the slight partition of iso-propyl acetate and by-product iso-propanol into water. The base was added at a rate to give a post-separation consistent aqueous pH of 12.8. Iso-propyl acetate was fed to give a biphasic system for back-extraction of the (*S*)-amine into the organic phase, Schemes 1 and 2. Following the back-extraction the L–L phase separation was done using a Zaiput, as there were no further downstream pressure differentials. This allowed for the complete back-extraction of (*S*)-amine into the organic phase, as well as a 6% recovery of the (*R*)-amide previously lost.

Purification of the (*S*)-amine was achieved by salt formation and crystallisation with *p*-TsOH in 2-MeTHF, eliminating any residual (*R*)-amide. The organic phase containing (*S*)-amine and acid were co-fed into a stirred beaker give (*S*)-amine. *p*-TsOH salt, causing its crystallisation from solution, Schemes 1 and 2. The product amine salt was then isolated, using batch filtration and drying, as colourless crystals in 43% isolation yield (theory 49%) with >99% ee, whilst the (*R*)-amide co-product was obtained in 50% isolation yield (theory 51%) and 96% ee. The productivity of the system for production of the enantiopure salt was 285 g L^−1^ h^−1^.

## Conclusions

In summary, we have successfully achieved a green, novel continuous biocatalytic system for the synthesis, and in-line purification of amines using the recyclable and non-toxic biocatalyst N435. The N435 product stream can be effectively purified and separated by in-line pH-based extractions and crystallisation, giving a PMI of around 400 for the whole continuous system. These results show how the telescoping of continuous flow reactions through in-line purification (liquid–liquid extraction and crystallisation in this work) can open up parameter space to the chemist, such as the ability to scale-up flow systems, reduce residence times and improve sustainability. The methods could be applied to other catalytic reactions such as transaminase, dynamic kinetic resolution (DKR).^23,44^ Furthermore, the method indicates how the use of biocatalysts which require cofactors might be continuously separated and recycled for reducing cost, improving efficiency minimising waste.

## Data availability

The data supporting this article have been included as part of the ESI.†

## Conflicts of interest

There are no conflicts to declare.

## Supplementary Material

RE-010-D4RE00442F-s001
