# Supplementary material for: Integrating continuous flow reaction and work-up: chiral amine resolution, separation and purification using a novel coalescing filter system
Source: React Chem Eng. 2024 Nov 13;10(2):392–7. doi: 10.1039/d4re00442f (PMC11603407; doi:10.1039/d4re00442f)
Supplement: RE-010-D4RE00442F-s001 [file RE-010-D4RE00442F-s001.pdf]

Supplementary Information (SI) for Reaction Chemistry & Engineering.
This journal is © The Royal Society of Chemistry 2024

Electronic Supplementary Information (ESI) for

This journal is © The Royal Society of Chemistry 2024

Supporting Information

Continuous Flow Lipase Catalysed Resolution and In-line Purification of Amines using pH Controlled Extractions.

Bethan M. Rowley,[a] Luke A. Power,[a] Lisa A. Thompson,[a] Emma Parks,[b] James Birbeck,[b] Steve Marsden,[a] Nikil Kapur,[a] and John A. Blacker*[a]

a. Institute of Process Research and Development, School of Chemistry & School of Chemical and Process Engineering, University of Leeds, Leeds, LS2 9JT, United Kingdom.

b. Croda Europe Limited, Bridge Ln, Rowcliffe Bridge, Goole, DN14 8PN, United Kingdom.

Table of Contents

1. System Configuration 2
1.1 Set-up 2
1.2 Aluminium Heating Block and Packed-bed Reactor 3
1.3 Determination of Residence Time for Packed-bed Reactor 4
1.4 24-Hour Stability Test 5
2. Procedures 5
2.1 General Considerations 5
2.2 Experimental Procedure 5
2.3 Chiral GC analysis 6
2.4 Process Mass Intensity 8
3. Results 9
3.1 N435 Catalysed Resolution 9
3.2 Acid-Extraction of 1-phenylethylamine 9
3.3 pH-Based Extractions 9
3.3 Synthesis of (*S*)-1-phenylethanaminium 4-methylbenzenesulfonate 10
3.4 Synthesis of *rac*-*N*-(1-phenylethyl)acetamide 10
3.5 Synthesis of *S*-*N*-(1-phenylethyl)acetamide 11
3.5 Synthesis of *R*-*N*-(1-phenylethyl)acetamide 11
4. Data for Synthesised Compounds 12
4.1 (*S*)-1-phenylethanaminium 4-methylbenzenesulfonate 12
4.3 (*S*)-*N*-1-phenylethylacetamide 14
4.4 (*R*)-*N*-1-phenylethylacetamide 14

References 15

1. System Configuration

1.1 Set-up

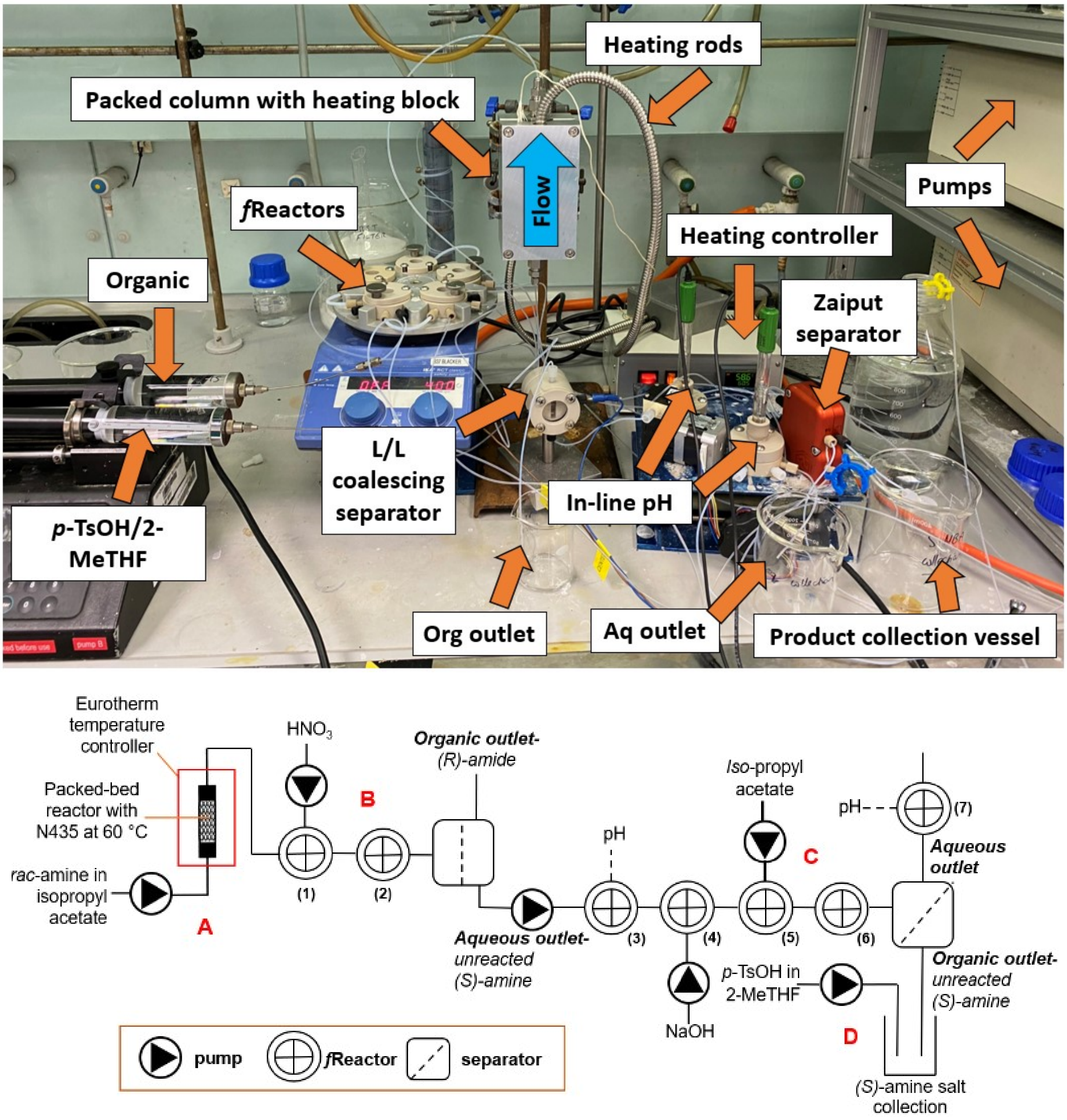

Figure 1: Configuration of continuous flow system for N435 resolution A, acid-extraction B, back-extraction C and crystallisation D.

Reservoir solutions were prepared by mixing the desired reagents with solvent under ambient conditions. Reaction mixture solution was prepared with *rac*-1-phenylethylamine (*rac*-1) (2.13 g; 17.5 mmol; 0.07 M), biphenyl (0.77 g, 0.02 M) as the internal standard and made up with isopropyl acetate (250 mL). The acid reservoir was prepared with nitric acid (69% w/w; 3.24 mL; 0.05 M) and made to 1000 mL with deionised water. The base reservoir was prepared with sodium hydroxide (80.06 g, 2.00 M) and sodium chloride (60.0 g) and made up with deionised water (1.00 L). Organic reservoir solution was prepared with isopropyl acetate and biphenyl (0.31 g, 0.02 M) used as the internal standard. The

crystallisation reservoir slution was prepared with p-toleunesulfonic acid (0.60 g, 3.50 mmol, 0.035 M) and made up with 2-MeTHF (100 mL).

The system was set up in the manner detailed in Figure 1. An initial packed-bed reactor (PBR) made from steel tubing (length: 113 mm, inner dia: 4.5 mm) was encased in an aluminum heating block and heated to 60 °C controlled by a Eurotherm temperature controller, where the inlet of the reaction mixture was pumped from the bottom of the packed-bed reactor. An initial fReactor with a faceplate mount had one port closed with a plug nut. Two ports were left to attach the acid inlet and the resultant packed-bed stream and a final was attached to the second standard fReactor. The second fReactor had two ports closed with one port for the inlet from the previous fReactor and the other for the outlet stream. This outlet carried the biphasic mixture to the L/L coalescing separator.[1] The organic outlet from the separator was left for sampling for the (*R*)-amide (*R*-2) and the aqueous outlet was attached to a probe holder fReactor for pH analysis. Two ports of this were closed, one was for the inlet of the aqueous stream and the other was the outlet to the next set of fReactors.

The third fReactor had one port closed with a plug nut. Two ports were left to attach the base inlet and the previous aqueous stream, and a final was attached to the fourth standard fReactor. The fourth fReactor also had one port closed, where two ports were left to attach the organic inlet and the previous aqueous mixture. The final port was attached to the final fReactor which has two ports closed, with one port from the previous biphasic mixture and the other for the outlet stream. This outlet carried the biphasic mixture to the Zaiput membrane separator.[2] The aqueous outlet from the separator was attached to a probe holder fReactor for pH analysis and the organic outlet flowed into a product collection vessel. PTSA in 2-MeTHF was also pumped into the product collection vessel where it met with the organic and consequently crashed the unreacted (*S*)-amine (*S*-1) out of solution.

The set-up for the in-line pH measurements was previously demonstrated by Power *et al.*[3] The development of the L/L coalescing separator was previously designed by Daglish *et al.*[1]

1.2 Aluminium Heating Block and Packed-bed Reactor

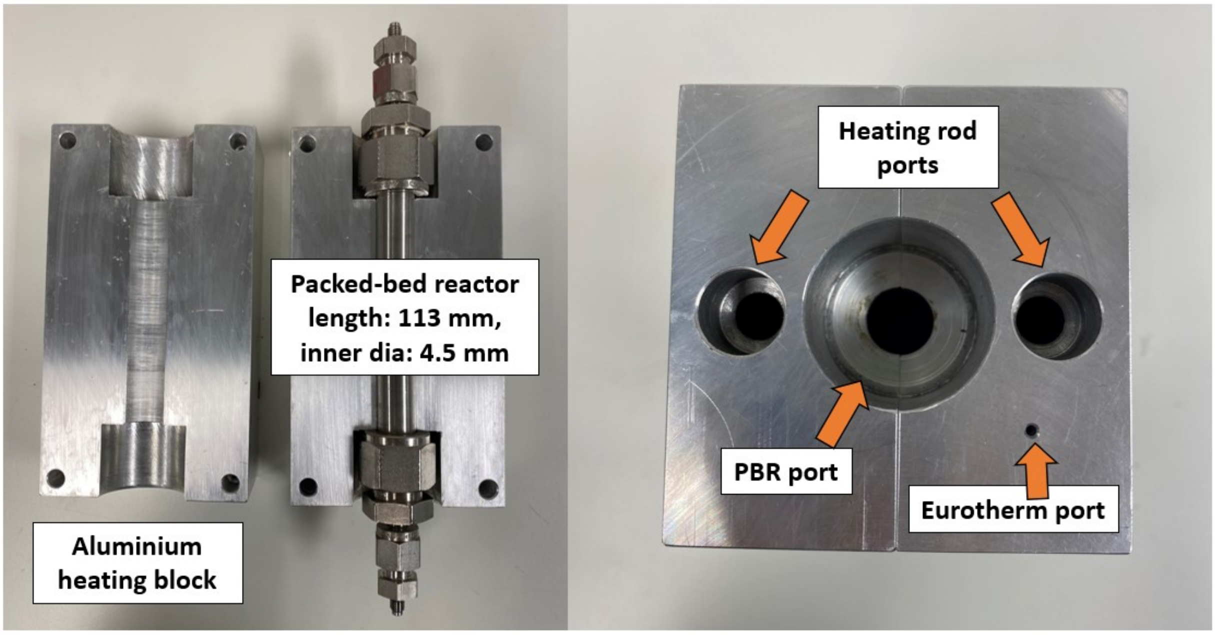

Figure 2: Ports and assembly of aluminium heating block and packed-bed reactor. Left: Inside heating block and placement of packed-bed reactor. Right: Bespoke port holes made for Eurotherm, heating rods and packed-bed reactor.

In order to heat the PBR and maintain it at 60 °C, a bespoke aluminium heating block was made to fit the exact size of the PBR. The heating block had two holes made to fit the heating rods so it could heat up either side of the aluminium block surrounding the PBR. The heating rods were controlled by a Eurotherm temperature controller in which a small hole was made so the Eurotherm could measure the temperature inside of the aluminium block and alter the temperature if needed. This allowed for a constant temperature of 60 °C to remain steady throughout the duration of the continuous system.

1.3 Determination of Residence Time for Packed-bed Reactor

In order to determine the residence time for the flow reaction, the void space in the PBR was calculated as shown below.

Calculation method based on volume of filled solvent:

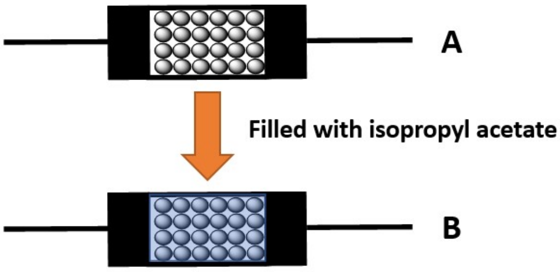

The weight of the PBR is measured (enzyme and glass beads)

A = 237.61 g

Isopropyl acetate (iPrOAc) is passed through the PBR and the weight is measured (enzyme, glass beads and iPrOAc)

B = 243.40 g

The volume of iPrOAc (= void space) is calculated by using the density of iPrOAc

Void space = Volume of iPrOAc = [(B g) – (A g)] / 0.87 g/cm^3 (density of iPrOAc)

= 6.70 cm^3 = 6.70 mL

Adopting 6.70 cm^3 as reaction volume for calculating residence time, residence time equals 6 min in the case of 1.12 mL/min as total flow rate.

1.3.1 Residence Time Screen

To be able to determine which residence time for the N435 resolution gave the highest conversion to (*R*)-amide (*R*-2) and highest enantiomeric excess (*ee*), different residence times were explored, as shown below:

Table 1: Exploration of residence times and corresponding R-amide yields and ee. Based on a 6.7 mL PBR.

Residence Time	Flow rate (mL/min)	*R*-2 conversion (%)	*R*-2 *ee* (%)
3	2.23	40	96
**6**	**1.12**	**44**	**>99**
10	0.67	35	87
15	0.45	35	82
30	0.22	38	90

A residence time of 6 minutes (flow rate: 1.12 mL/min) gave the highest conversion to *R*-2 (44% from *rac*-1) and the highest *ee* (100%) so was used in the continuous flow platform.

1.4 24-Hour Stability Test

A stability test was carried out over a 24-hour period to test the longevity of enzyme activity, as shown below:

Table 2. 24-hour stability test for the N435 resolution of 1-phenylethylamine.

Time (hour)	*R*-2 conversion (%)	*R*-2 *ee* (%)
0	40	>99
1	40	>99
2	41	>99

3	42	>99
18	40	>99
19	39	>99
20	40	>99
21	39	>99
22	40	>99
23	39	>99
24	39	>99

Results showed no drop off in enzyme activity and remained consistent over the 24-hour period. The PBR (vol. 6.2 mL) was filled with 1g N435 and 6.1g glass beads (dia. 0.5 mm), and the reaction mixture made up of 0.07 M 1-phenylethylamine in *iso*propylacetate was pumped through the PBR at a rate of 1.03 mL/min (6 min tRes).

2. Procedures

2.1 General Considerations

All the following compounds were purchased from suppliers and used without further purification. 1-phenylethylamine (99%), biphenyl (99%), sodium hydroxide (NaOH, pellets), sodium chloride (NaCl, extra pure), *p*-toluenesulfonic acid monohydrate (*p*-TsOH, 99%, extra pure), (*S*)-(-)-1-phenylethylamine (99% *ee*), (*R*)-(-)-1-phenylethylamine (98% *ee*) and 2-methyltetrahydrofuran (2-MeTHF, >99.5%) were purchased from Fisher Scientific Ltd. Isopropyl acetate (98%) and nitric acid (HNO_3, 69%) were purchased from Merck Life Science UK Ltd. Lipase acrylic resin from candida antartica (N435, >=5000 U/g, recombinant, expressed in Aspergillus niger) was purchased from Scientific Laboratory Supplies. Melting point analysis was performed on a Stuart SMP10 melting point apparatus. Optical resolution analysis was carried out on a Polartronic H532 Polarimeter. FT-IR spectroscopy was performed on a Bruker ALPHA Platinum-ATR FT-IR Spectrometer. NMR spectroscopy was performed on a Bruker 400 AVANCE III HD NMR Spectrometer (*1*H NMR at 400 MHz and *13*C NMR at 101 MHz) with the appropriate deuterated solvent. Chemical shifts in *1*H and *13*C NMR spectra are expressed as ppm downfield from TMS, and reported as singlet (s), doublet (d), triplet (t), quartet (q) and combination thereof, or multiplet (m). Chiral GC analysis was carried out on an Agilent HP 6890 instrument fitted with a CP Chirasil-Dex-CB column (25 m x 0.25 mm, 0.25 µm film thickness), H_2 carrier gas, FID detector.

2.2 Experimental Procedure

The system was setup as mentioned in section 1.1. All pump inlets were kept constant at 1.12 mL/min. the hotplate was set to 400 rpm. Samples and pH readings were taken at various reactor volumes after steady state was reached after 1440 seconds, and when the pH after extraction had reached a constant. Reservoir solutions were prepared by dissolving the desired reagents in solvent under stirring at ambient conditions.

1-phenylethylamine (2.22 g, 0.07 M) and biphenyl (0.77 g, 0.02 M) as the internal standard in isopropyl acetate (250 mL) was pumped (1.12 mL/min) using a JASCO PU-980 dual piston HPLC pump into a steel packed-bed reactor (volume: 6.7 mL, length: 113 mm, Inner Diameter: 4.5 mm) filled with N435 (2.00 g) and glass beads (0.5 mm, 1.87 g). The packed-bed reactor was connected to Swagelok SS810-R4 and

SS-400-6-1ZV reducers and encased in a bespoke aluminium heating block and heated with a Eurotherm 3200 temperature controller at 60 °C. The resultant product stream flowed into sequential PEEK fReactor CSTRs and containing a PTFE cross-shaped magnetic stir bar. HNO_3 (0.05 M) was pumped (1.12 mL/min) using a JASCO PU-980 dual piston HPLC pump into the PEEK fReactor CSTRs. The biphasic reaction mixture was separated using a L/L coalescing separator fitted with PBT filter and the aqueous phase pumped using a KNF FEM 1.02 TTSM-2 pump into a PEEK chamber fitted with a Hanna Instruments HI-14132B pH probe. NaOH (2 M) with NaCl (~60 g) was pumped (1.12 mL/min) using a JASCO PU-980 dual piston HPLC pump into PEEK fReactor CSTRs along with resultant aqueous stream. A mixture of biphenyl (0.31 g, 0.02 M) in isopropyl acetate (100 mL) was drawn into in a 50 mL SGE gastight Luer lock syringe was pumped (1.12 mL/min) using a Harvard apparatus model 11 syringe pump into PEEK fReactor CSTRs containing a PTFE cross-shaped magnetic stir bar. The biphasic reaction mixture was separated using a Zaiput technologies SEP-10 L/L separator fitted with an O/B 900 membrane and the resultant aqueous phase flowed into PEEK chamber fitted with a Hanna Instruments HI-14132B pH probe. PTSA (0.6029 g, 0.035 M) in 2-MeTHF (100 mL) was drawn into a 50 mL SGE gastight Luer lock syringe was pumped (1.12 mL/min) using Harvard apparatus model 11 syringe pump into product collection vessel. The resultant amine was collected by vacuum filtration to obtain (*S*)-1-phenylethanaminium 4-methylbenzenesulfonate as colourless crystals (0.29 g, 0.98 mmol, 43%).

2.3 Chiral GC analysis

Unless otherwise stated, all reactions were analysed by chiral GC to determine conversion and enantiomeric excess (*ee*) in comparison to biphenyl used as the internal standard. The GC oven method used: 65 °C (hold for 3 min); 0.5 °C/min to 80 °C (hold for 20 min); 40 °C/min to 180 °C (hold for 10 min); Inj = 300 °C; Det = 300 °C; H_2 carrier gas; 15 psi.

Retention times: *R*-amine: 23.47 min, *S*-amine: 23.89 min, *S*-amide product (minor): 56.72 min, *R*-amide product (major): 56.88 min.

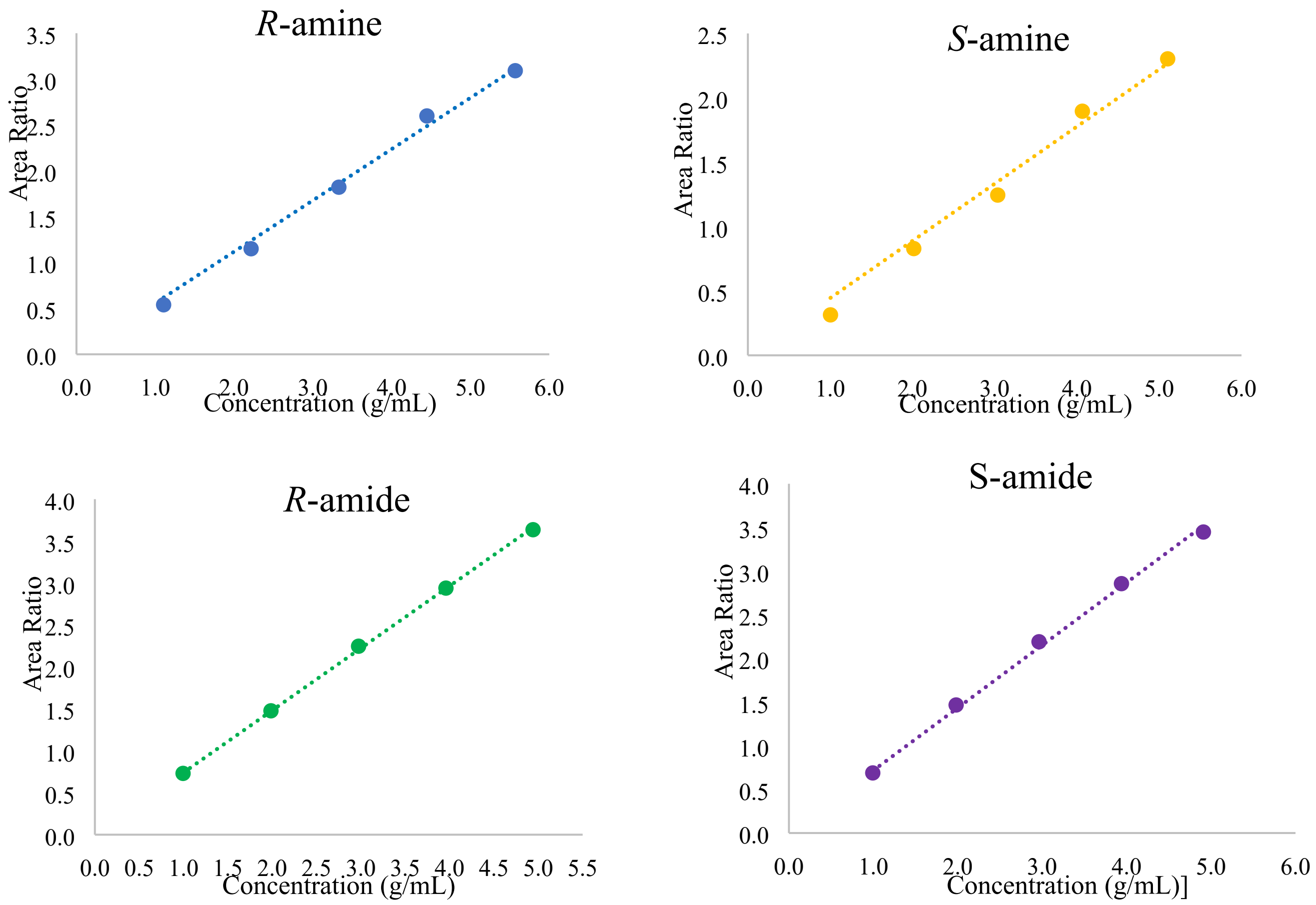

Figure 3. Calibration data for R/S amine and R/S amide in comparison to the internal standard (biphenyl).

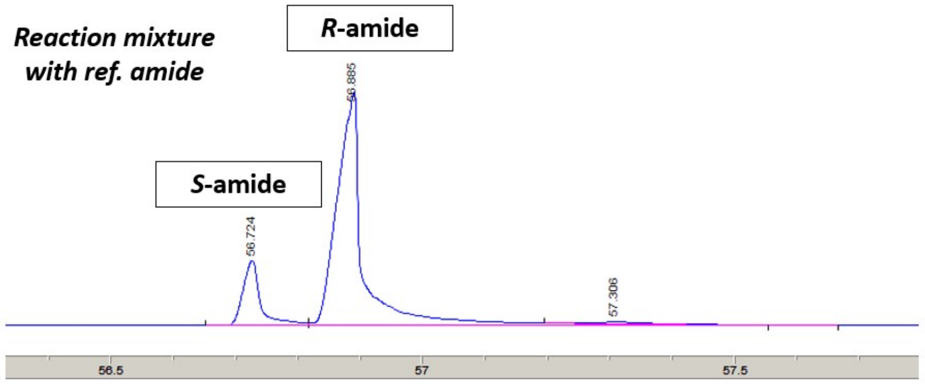

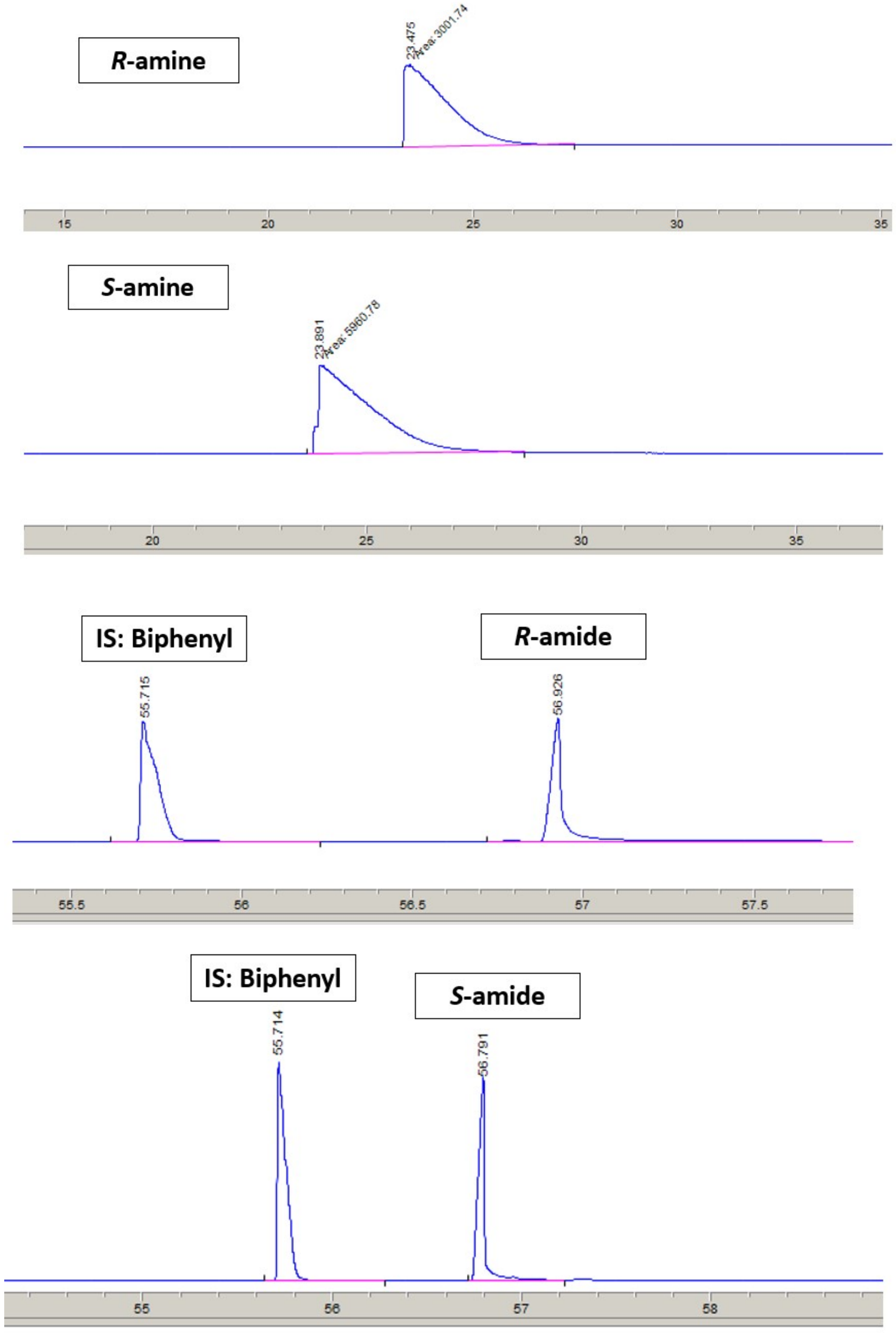

Figure 4: GC data of reaction mixture with racemic amide reference, R/S amine and R/S amide with internal standard.

Conversion of *R*-amide from *rac*-1 was calculated via below:

$$Conc.\,of\ R - amide\ (M)\ = \frac{\frac{R - amide\ area}{IS\ area} \times conc.\,of\ IS}{m}$$

$$Conversion\ (\%)\ = \frac{Conc.\,of\ R - amide}{Conc.\,of\ reaction\ mixture} \times 100$$

Where *m* is from the equation of a line present in the calibration curves, shown in Figure 3.

The enantiomeric excess (*ee*) of *R*-amide was calculated using the following equation:

$$ee = \frac{area\ of\ R - amide - area\ of\ S - amide}{area\ of\ R - amide + area\ of\ S - amide} \times 100$$

Where the area of *R*-amide has a larger peak area over *S*-amide.

2.4 Process Mass Intensity

Process mass intensity (PMI) is the key mass-based green metric used to measure the greenness of manufacturing processes and is used to help improve the efficiency of sustainable processes within industry.[4] The process mass intensity (PMI) value can be calculated as the amount of reagents, reactants, catalyst and solvent relative to the amount of isolated product as shown as the following equation:[5]

$$PMI = \frac{mass_{reactants} + mass_{reagents} + mass_{catalyst} + mass_{solvent}}{mass\ of\ isolated\ prouct}$$

$Mass_{reactants}$ = 0.55 g (*rac*-1-phenylethylamine)*

$Mass_{reagents}$ = 0.39 g (*p*-TsOH)*

$Mass_{catalyst}$ = 2.00 g (N435)

$Mass_{solvent}$ = 56.7 g (iPrOAc)*, 55.9 g (2-MeTHF)*

Mass of isolated product = 0.29 g

* mass based off volume of reaction mixture used within reaction (65 mL)

$$PMI = \frac{0.55 + 0.39 + 2.00 + 112.6}{0.29}$$

$$PMI = 398.4$$

3. Results

3.1 N435 Catalysed Resolution

Table 3: Results for N435 catalysed resolution.

Reactor Volumes	*R*-2 yield (%)	*ee* of *R*-2 (%)
4	50	96
6	50	96
8	50	96

Before introducing the pH-based extractions and in-line crystallisation, the intial resolution was sampled at 4 RVs after steady-state had been reached and ran for a further 4 RVs. The results showed full conversion of *R*-2 (50% from *rac*-1) and high enantiomeric excess (96% *ee*).

3.2 Acid-Extraction of 1-phenylethylamine

The acid-extraction of 1-phenylethylamine in toluene was achieved using a varying concentration of HCl (0-1 M). The separation of the biphasic mixture was carried out using both a Zaiput membrane separator as well as the L/L coalescing separator to allow for comparison. Results for the extraction efficiency of 1-phenylethylamine is shown below:

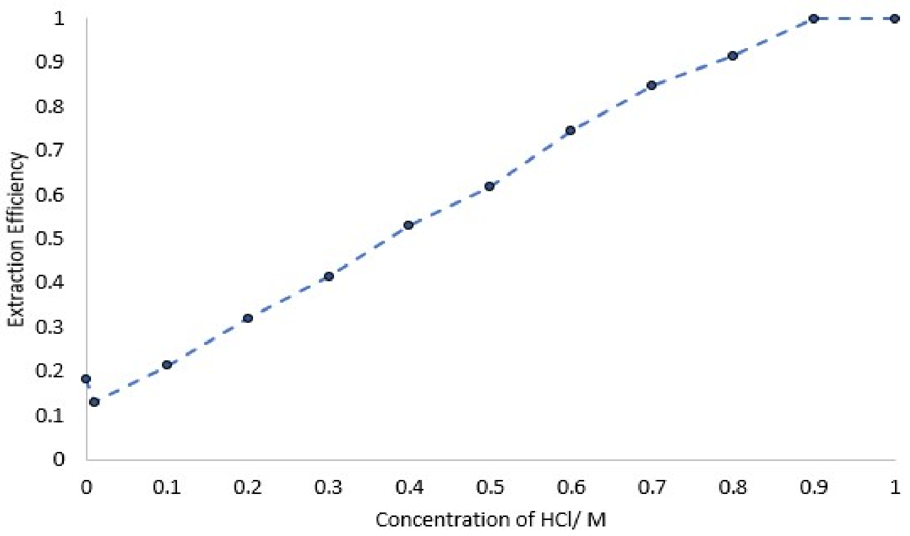

Figure 5. The extraction efficiency of 1-phenylethylamine compared to the inlet acid concentration ranging from 0 to 1 M.

3.3 pH-Based Extractions

Table 4: Results for acid-extraction and back-extraction.

Reactor Volumes	*R*-2 yield (%)	*ee* of *R*-2 (%)	pH
After acid-extraction and separation			
4	41	96	1.46
6	40	96	1.45
8	40	96	1.42
After back-extraction and separation			
4	6	-	12.87
6	6	-	12.88
8	7	-	12.83

After the acid-extraction and separation, the conversion of *R*-2 decreased slightly (41% from *rac*-1) due to the slight miscability of isopropyl actetate and water, which caused some of the *R*-2 present in the organic phase to transfer across to the aqueous phase. The enantiomeric excess for *R*-2 remained unaltered (96% *ee*). The pH of the aqueous stream after separation also remained at a constant of 1.4. At this stage, the unreacted *S*-amine had been protonated and transferred across to the aqueous phase. After the back-extraction and separation, the results showed the recovery of *R*-2 as some of the *R*-2 which transferred across to the aqueous phase had been back-extracted into the organic phase. The pH of the aqueous stream after separation remained at a constant of 12.8. At this stage, the unreacted *S*-amine present in the aqueous phase had been deprotonated back into the organic phase, available for the in-line crystallisation of *S*-amine out of solution with *p*-TsOH, giving the *S*-amine salt in a high yield of 43% out of a possible 50%.

3.3 Synthesis of (*S*)-1-phenylethanaminium 4-methylbenzenesulfonate

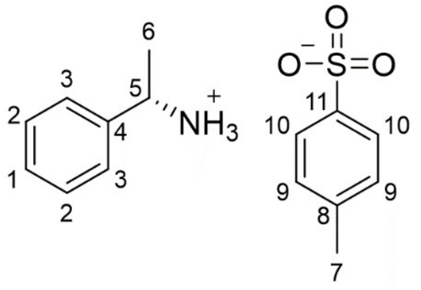

Para-toluenesulfonic acid (0.60 g, 0.035 M) dissolved in 2-methyltetrahydrofuran (100 mL) was pumped (1.12 mL/min, 44.8 mL) into a collection vessel where it met with the previous product stream containing *S*-1-phenylethylamine in isopropyl acetate. After 48 minutes the in-line crystallisation was stopped and the product collected *via* vacuum filtration and (*S*)-1-phenylethanaminium 4-methylbenzenesulfonate was isolated as colourless crystals (0.29 g, 0.98 mmol, 43%). mp 157-159 °C; $[\alpha]_D^{25}$ = -1.62° (*c* = 0.05, MeOH); FT-IR (ν cm^{-1}, ATR): 2105 (S=O), 1530 (N-H); ^1H NMR (D_2O, 400 MHz, *δ* ppm): 1.65 (d, *J* = 6.8 Hz, 3H, CH_3), 2.40 (s, 3H, CH_3) 4.53 (q, *J* = 6.8 Hz, 1H, CH), 7.37 (d, *J* = 8.0 Hz, 2H, ArH), 7.45-7.52 (m, 5H, ArH), 7.69 (d, *J* = 8.4 Hz, 2H, ArH); ^{13}C NMR (D_2O, 100.61 MHz, *δ* ppm): 19.3 (6-CH_3), 20.4 (7-CH_3), 60.0 (C5), 125.3 (C10), 126.4 (C1), 129.1 (C2), 129.2 (C3), 129.4 (C9), 137.6 (C8), 139.4 (C4), 142.4 (C11). Data obtained was similar with existing literature for *R*-enantiomer.[6]

3.4 Synthesis of *rac*-*N*-(1-phenylethyl)acetamide

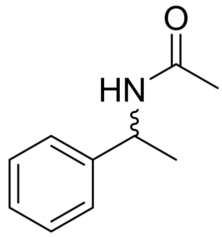

Triethylamine (1.20 mL, 8.60 mmol) was added to *rac*-1-phenylethylamine (1.01 g, 8.33 mmol) in dichloromethane (20.0 mL). The mixture was cooled on ice and acetic anhydride (1.00 mL, 10.6 mmol) was added dropwise and the reaction stirred at room temperature for 2.5 h. Water (20.0 mL) was then added and the layers separated. The organic phase was washed with HCl (1.00 M, 3 x 10.0 mL), dried (Na_2SO_4) and the solvent removed under vacuum to give *rac*-*N*-(1-phenylethyl)acetamide as a pale orange solid (0.88 g, 5.40 mmol, 65.0%). ^1H NMR ($CDCl_3$, 400 MHz, *δ* ppm): δ 1.41 (d, *J* = 4.0 Hz, 3H, $CHCH_3$), 1.90 (s, 3H, C(O)CH_3), 5.05 (m, *J* = 7.3 Hz, 1H, $CHCH_3$), 5.79 (br s, 1H, NH), 7.16 – 7.31 (m, 5H, Ar-H); *m/z* (ESI^+): [M+H]+ 163.74; Chiral GC analysis using standard method described in Section 2.3: t_R(N-acetyl-[(1*S*)-1-phenylethyl]amine) = 56.86 min, t_R(N-acetyl-[(1*R*)-1-phenylethyl]amine) = 57.08 min. Data obtained was consistent with existing literature.[7]

3.5 Synthesis of *S*-*N*-(1-phenylethyl)acetamide

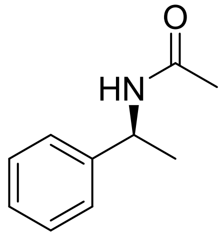

(*S*)-(-)-1-phenylethylamine (1.02 g, 8.42 mmol) was dissolved in dichloromethane (7.50 mL) and the mixture was cooled on ice. Acetic anhydride (0.65 mL, 6.88 mmol) was added dropwise and the reaction stirred at room temperature for 1.0 h. $NaHCO_3$ (25.0 mL) was then added and the layers separated. The organic phase was washed with water (3 x 10.0 mL), dried (Na_2SO_4) and the solvent removed under vacuum to give *S*-*N*-(1-phenylethyl)acetamide as a white solid (0.84 g, 5.15 mmol, 75.0%). ^{1}H NMR ($CDCl_3$, 400 MHz, δ ppm): δ 1.49 (d, *J* = 6.8 Hz, 3H, $CHCH_3$), 1.98 (s, 3H, C(O)CH_3), 5.09-5.18 (m, 1H, $CHCH_3$), 5.67-5.75 (br s, 1H, NH), 7.23-7.37 (m, 5H, Ar-H); *m/z* (ESI$^+$): [M+H]+ 163.74; Chiral GC analysis using standard method described in Section 2.3: t_R(N-acetyl-[(1*S*)-1-phenylethyl]amine) = 56.73 min. Data obtained was consistent with existing literature.[7]

3.5 Synthesis of *R*-*N*-(1-phenylethyl)acetamide

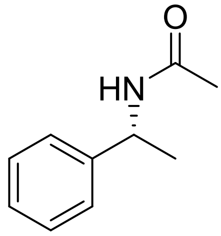

(*R*)-(-)-1-phenylethylamine (1.04 g, 8.58 mmol) was dissolved in dichloromethane (7.50 mL) and the mixture was cooled on ice. Acetic anhydride (0.60 mL, 6.35 mmol) was added dropwise and the reaction stirred at room temperature for 2.0 h. $NaHCO_3$ (25.0 mL) was then added and the layers separated. The organic phase was washed with water (3 x 10.0 mL), dried (Na_2SO_4) and the solvent removed under vacuum to give *R*-*N*-(1-phenylethyl)acetamide as a white solid (0.85 g, 5.20 mmol, 82.0%). ^{1}H NMR ($CDCl_3$, 400 MHz, δ ppm): δ 1.49 (d, *J* = 6.0 Hz, 3H, $CHCH_3$), 1.98 (s, 3H, C(O)CH_3), 5.09-5.17 (m, 1H, $CHCH_3$), 5.65-5.74 (br s, 1H, NH), 7.23-7.37 (m, 5H, Ar-H); *m/z* (ESI$^+$): [M+H]+ 163.74; Chiral GC analysis using standard method described in Section 2.3: t_R(N-acetyl-[(1*R*)-1-phenylethyl]amine) = 56.89 min. Data obtained was consistent with existing literature.[7]

4. Data for Synthesised Compounds

4.1 (*S*)-1-phenylethanaminium 4-methylbenzenesulfonate

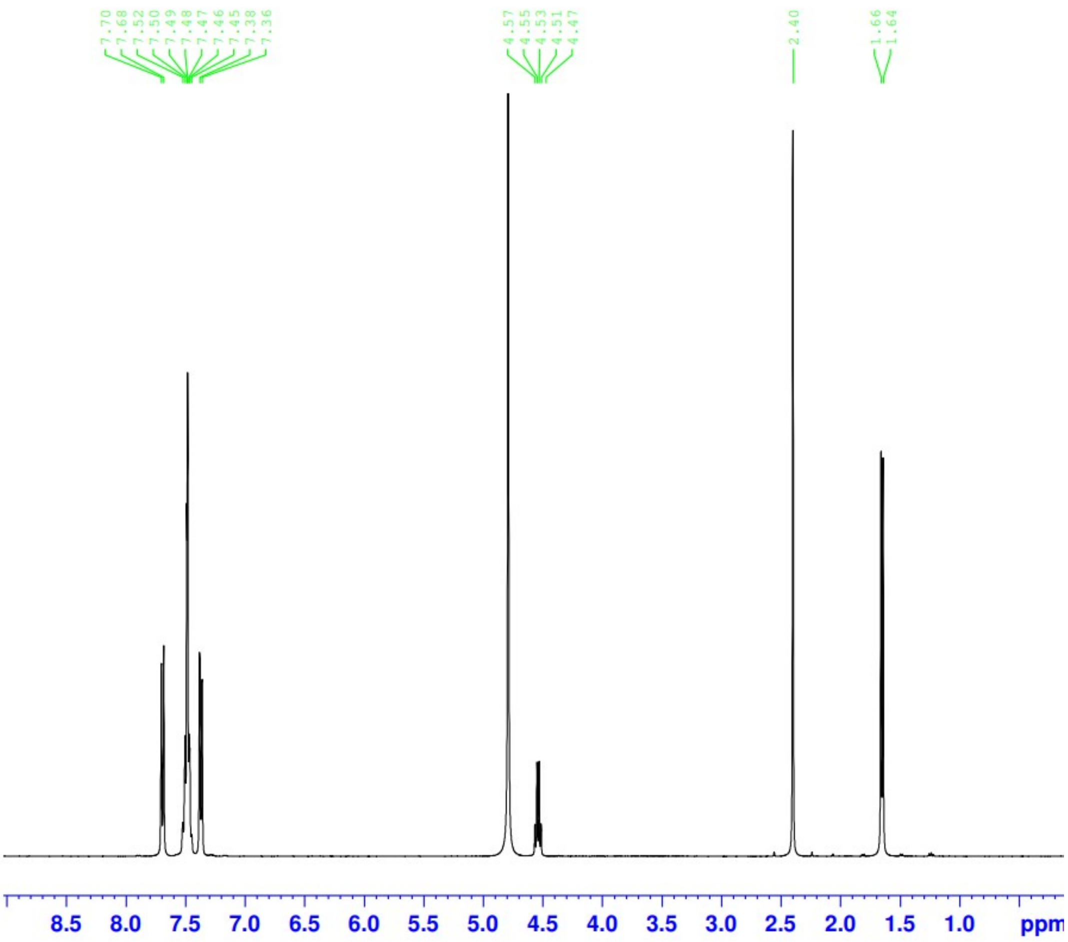

^{1}H-NMR spectrum (D_2O, 400 MHz) of (*S*)-1-phenylethanaminium 4-methylbenzenesulfonate

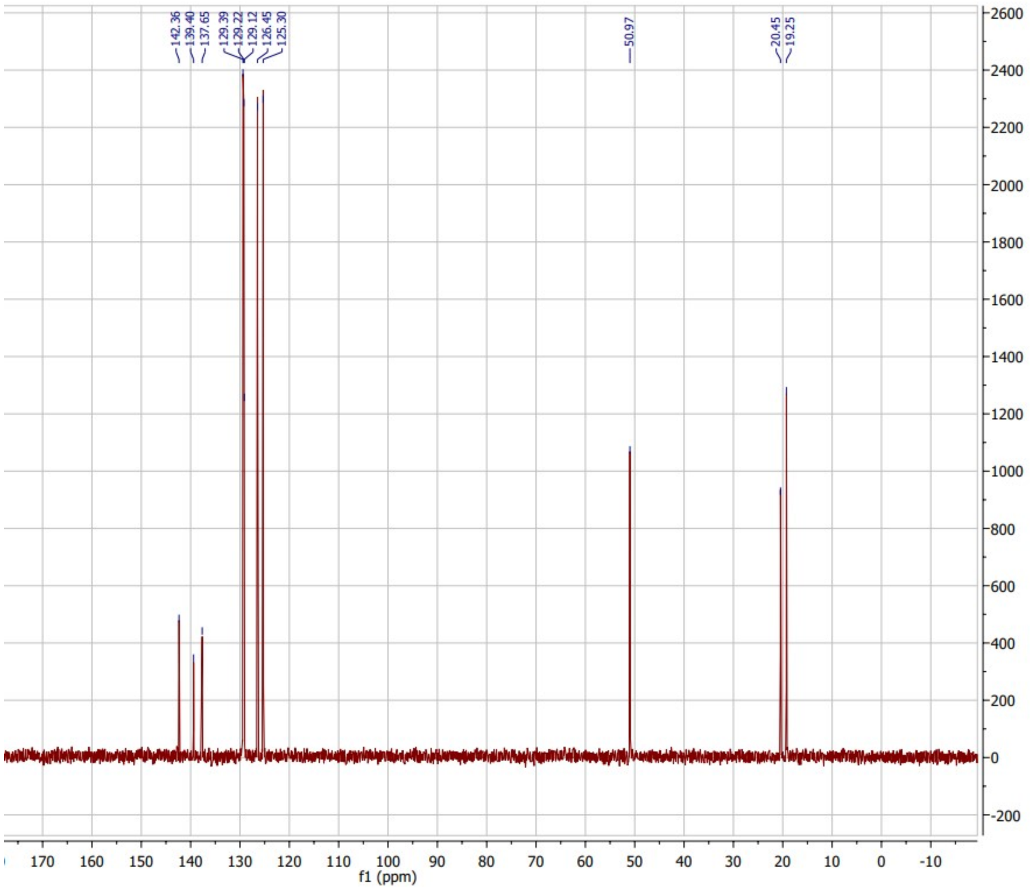

^{13}C-NMR spectrum (D_2O, 100 MHz) of (*S*)-1-phenylethanaminium 4-

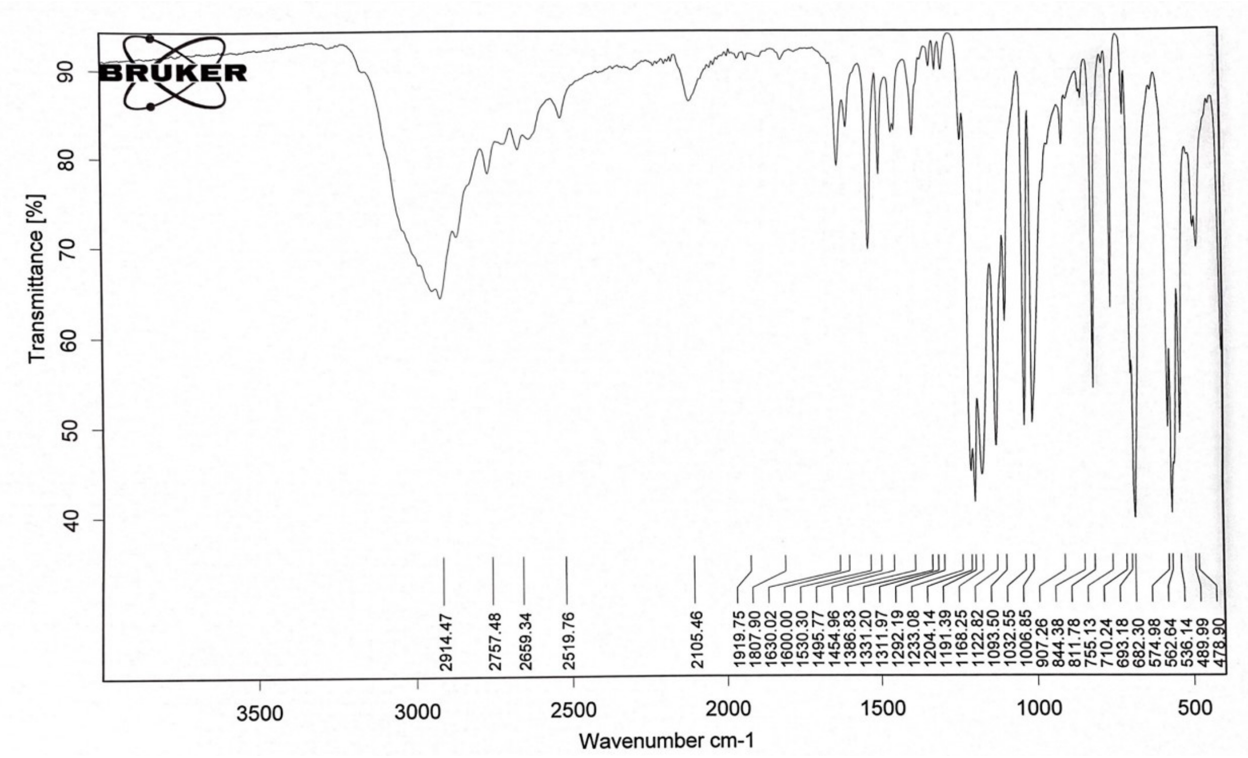

methylbenzenesulfonate

FT-IR spectrum (ν cm^{-1}, ATR) of (*S*)-1-phenylethanaminium 4-methylbenzenesulfonate

4.3 (*S*)-*N*-1-phenylethylacetamide

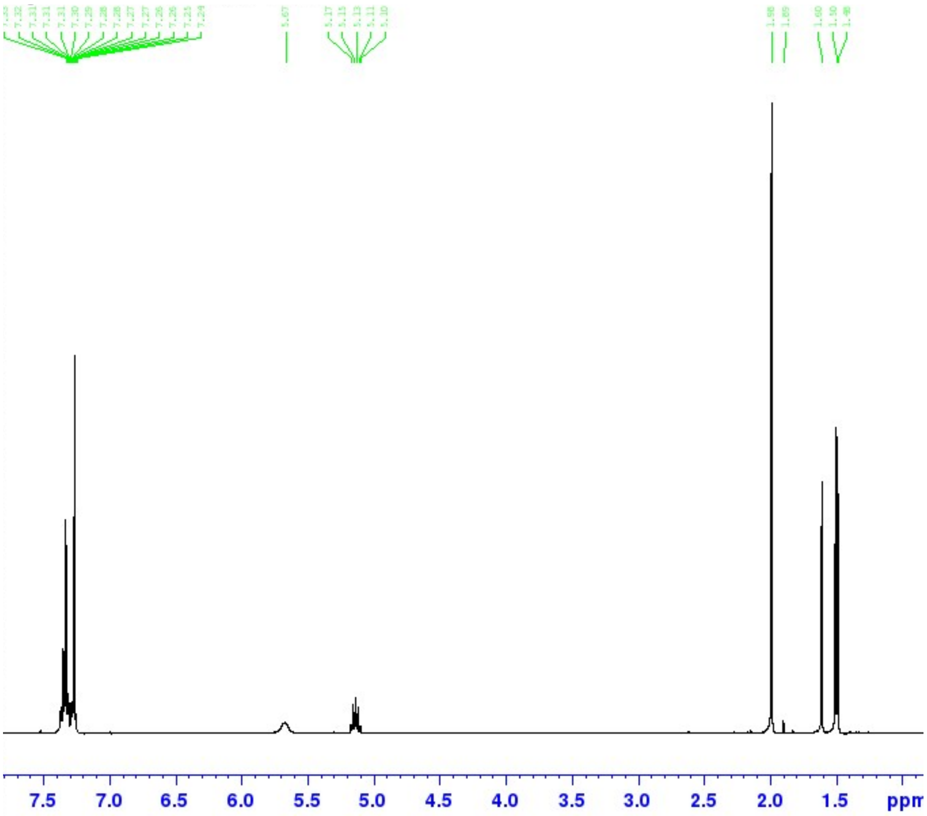

^{1}H-NMR spectrum (D_2O, 400 MHz) of (*S*)-1-phenylethylacetamide

4.4 (*R*)-*N*-1-phenylethylacetamide

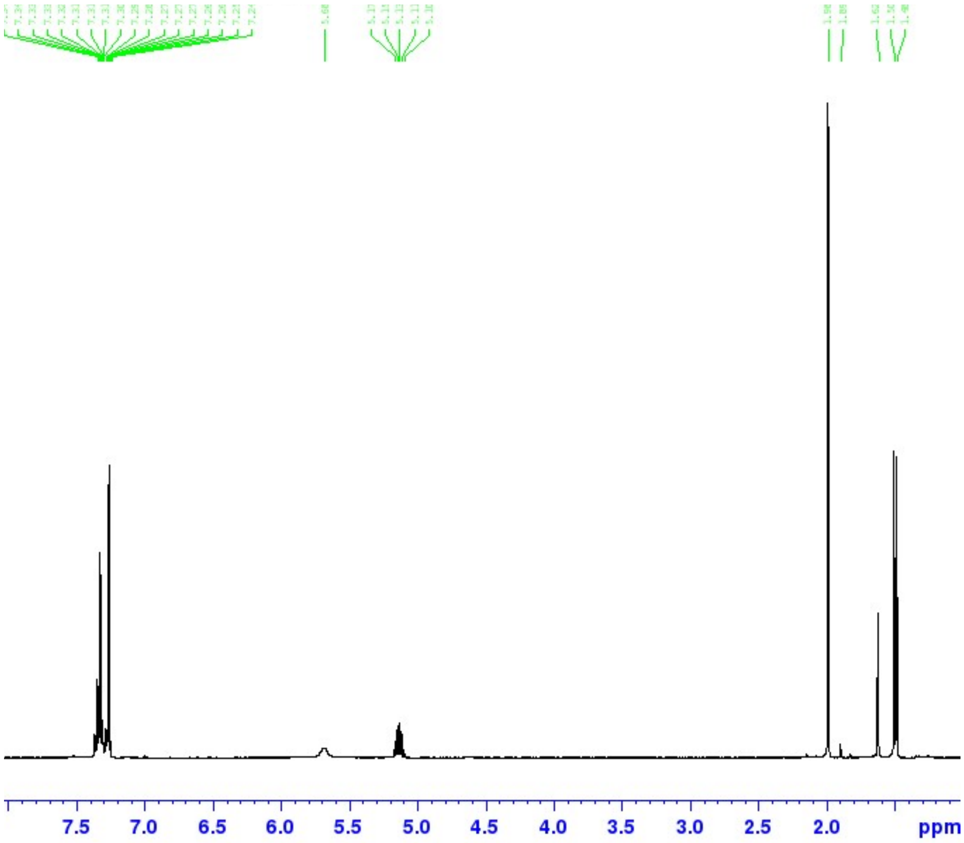

^{1}H-NMR spectrum (D_2O, 400 MHz) of (*R*)-1-phenylethylacetamide

References

1. Daglish, J.; Blacker, A. J.; de Boer, G.; Russell, S. J.; Tausif, M.; Hose, D. R. J.; Parsons, A. R.; Crampton, A.; Kapur, N., A Coalescing Filter for Liquid–Liquid Separation and Multistage Extraction in Continuous-Flow Chemistry. *Organic Process Research & Development* **2024,** *28* (5), 1979-1989.
2. https://www.zaiput.com/product/liquid-liquid-gas-separators/ Zaiput. (accessed 06/04/2022).
3. Power, L. A.; Clayton, A. D.; Reynolds, W. R.; Hose, D. R. J.; Ainsworth, C.; Chamberlain, T. W.; Nguyen, B. N.; Bourne, R. A.; Kapur, N.; Blacker, A. J., Selective separation of amines from continuous processes using automated pH controlled extraction. *Reaction Chemistry & Engineering* **2021,** *6* (10), 1806-1810.
4. Jimenez-Gonzalez, C.; Ponder, C. S.; Broxterman, Q. B.; Manley, J. B., Using the Right Green Yardstick: Why Process Mass Intensity Is Used in the Pharmaceutical Industry To Drive More Sustainable Processes. *Organic Process Research & Development* **2011,** *15* (4), 912-917.
5. Monteith, E. R.; Mampuys, P.; Summerton, L.; Clark, J. H.; Maes, B. U. W.; McElroy, C. R., Why we might be misusing process mass intensity (PMI) and a methodology to apply it effectively as a discovery level metric. *Green Chemistry* **2020,** *22* (1), 123-135.
6. Rajegowda, H. R.; Chethan, B. S.; Khan, R. u. R.; Lokanath, N. K.; Suchetan, P. A.; Kumar, P. R., Synthesis, crystal structure and thermal investigation of molecular salts of (R)-1-phenylethanamine combined with quantum chemical studies. *Journal of Molecular Structure* **2023,** *1272*, 134097.
7. Burk, M. J.; Wang, Y. M.; Lee, J. R., A Convenient Asymmetric Synthesis of α-1-Arylalkylamines through the Enantioselective Hydrogenation of Enamides. *Journal of the American Chemical Society* **1996,** *118* (21), 5142-5143.
